# Dent’s disease: An unusual cause of kidney failure 

**DOI:** 10.5414/CNCS110975

**Published:** 2023-01-12

**Authors:** Luís Leite de Sousa, Gonçalo Pimenta, Rita Veríssimo, Tiago J. Carvalho, Ivo Laranjinha

**Affiliations:** Nephrology Department, Hospital de Santa Cruz, Centro Hospitalar de Lisboa Ocidental, Carnaxide, Portugal

**Keywords:** CLCN5, Dent’s disease, hypercalciuria, low-molecular-weight proteinuria, nephrocalcinosis

## Abstract

Dent’s disease is an X-linked recessive disease characterized by proximal tubulopathy with low-molecular weight proteinuria, hypercalciuria, nephrolithiasis, nephrocalcinosis, and kidney failure. It is mainly caused by mutations in the *CLCN5* or *OCRL1* genes, and only ~ 250 families have been identified with these mutations. We present a 31-year-old male referred to a nephrology consultation due to elevated serum creatinine and a history of nephrolithiasis. Complementary evaluation revealed protein/creatinine ratio of 1.9 g/g and albumin/creatinine ratio of 0.5 g/g, hypercalciuria and medullary nephrocalcinosis. These findings raised the suspicion of Dent’s disease, which was confirmed by genetic testing. A missense mutation in the *CLCN5* gene (c.810C>G, p.(Ser270Arg)), not previously reported in populational databases, was identified. During the evaluation of the patient, it came to our attention that a first-degree male cousin was being followed in our kidney transplantation unit. Given the unknown etiology of his chronic kidney disease, genetic testing was performed, identifying the same mutation. This case highlights the importance of considering the diagnosis of Dent’s disease in the setting of a male patient with chronic kidney disease of unknown etiology, low-molecular-weight proteinuria, hypercalciuria, and nephrocalcinosis. Despite progression to end-stage kidney failure in a significant portion of male patients, there are no reports of recurrence after kidney transplantation.

## Introduction 

The concomitant presence of low-molecular-weight (LMW) proteinuria, hypercalciuria, nephrocalcinosis, nephrolithiasis, and kidney failure in a young individual should raise the suspicion of Dent’s disease, a rare disease mainly caused by mutations in *CLCN5* or *ORCL1* genes located on chromosome X [1]. These features are mainly identified in males, as soon as early childhood, while females may display a more subtle phenotype. We present a case of a patient and a direct relative who had already undergone kidney transplant due to chronic kidney disease (CKD) of unknown etiology, both carrying a mutation in the *CLCN5* gene not previously reported in populational databases. 

## Case report 

We report the case of a 31-year-old male with a history of nephrolithiasis – for which he was submitted for stone extraction – and a positive family history of nephrolithiasis (father). He was referred to a nephrology consult by his general practitioner due to a serum creatinine (SCr) of 2.4 mg/dL and microscopic hematuria. The physical examination was unremarkable, with normal blood pressure. 

**Figure 1.**
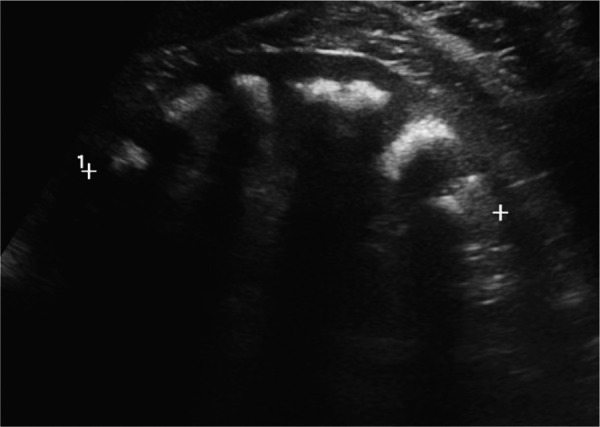
Kidney ultrasound showing medullary nephrocalcinosis with severe hyperechogenicity of the entire pyramids.

**Figure 2.**
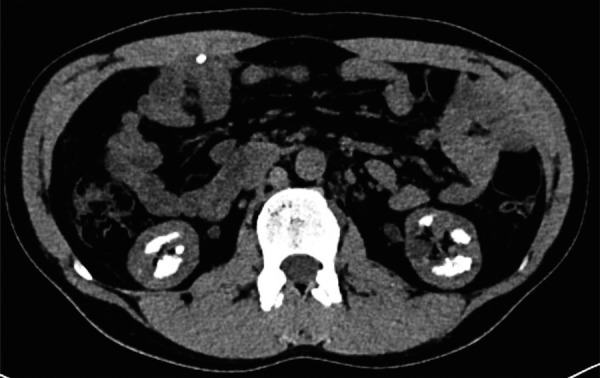
Abdomen CT (transversal view) showing medullary nephrocalcinosis.

**Figure 3:**
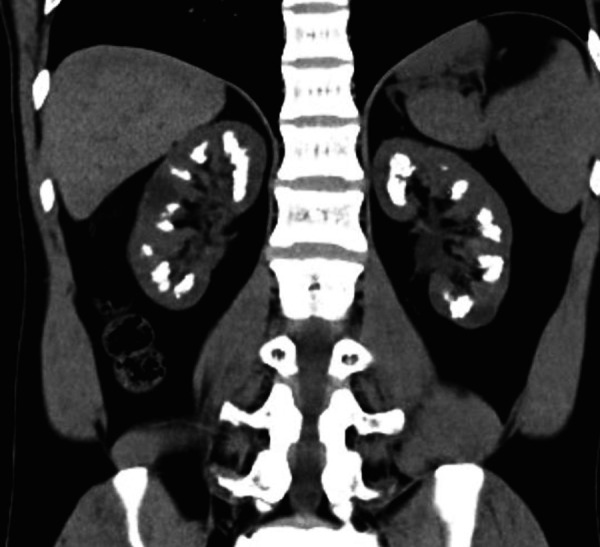
Abdomen CT (coronal view) showing medullary nephrocalcinosis.

The etiological study revealed a serum K^+^ 3.9 mmol/L (3.5 – 5.1 mmol/L), Ca2^+ ^10.0 mg/dL (8.6 – 10.0 mg/dL), phosphate 2.6 mg/dL (2.5 – 4.5 mg/dL), calcitriol 50 ng/mL (30 – 100 ng/mL), and iPTH 21 pg/mL (15 – 65 pg/mL). Urine evaluation showed pH of 7.0, microscopic hematuria, protein/creatinine ratio of 1.9 g/g with only 25% of albuminuria, higher urine calcium excretion (230 mg/24h; normal < 200 mg/24h), hypomagnesuria (65 mg/24h; 70 – 120 mg/24h), low urine chloride (80 mmol/24h; 110 – 250 mmol/24h). It also revealed a urinary excretion of sodium of 106 mmol/24h (40 – 220 mmol/24h), potassium of 70 mmol/24h (15 – 125 mmol/24h), oxalate of 0.29 mmol/24h (0.08 – 0.49 mmol/24h), citrate of 1.24 mmol/24h (0.6 – 4.8 mmol/24h), phosphate of 734 mg/24h (400 – 1,300 mg/24h), uric acid of 551 mg/24h (250 – 750 mg/24h), and no glycosuria. Arterial blood gas showed a serum pH of 7.34 and HCO3^–^ of 32 mmol/L. Kidney ultrasound revealed normal-sized kidneys, with reduced corticomedullary differentiation and medullary nephrocalcinosis (Figure 1, Figure 2, and Figure 3). These findings were confirmed by computed tomography scan. To ameliorate the hypercalciuria, the patient was started on potassium citrate and hydrochlorothiazide. One week later, thiazide was suspended due to symptomatic hypotension, worsening of kidney function, hypokalemia (3.3 mEq/L), and hypercalcemia (10.4 mg/dL). 

Given the constellation of findings (young male patient, kidney failure, microscopic hematuria, proteinuria with only 25% albumin, hypercalciuria, and medullary nephrocalcinosis), we considered the possibility of a syndrome of X-linked hypercalciuric nephrolithiasis. Genetic testing was performed at Genomed laboratory after extraction of DNA from peripheral blood. Genetic analysis was conducted through a search of anomalies in all the codifying regions and exon-intron junction regions (Twist human core exome plus RefSeq extension, Twist Bioscience, South San Francisco, CA, USA), followed by parallel massive sequencing (next-generation sequencing (NGS)) and confirmation by Sanger sequencing for regions not accurately analyzed by NGS. The missense variant c.810C>G, p.(Ser270Arg) in the *CLCN5* gene (NM_000084.4, chrX) in hemozigosity was detected. Although this variant is not reported in populational databases, it had been previously identified in an individual with Dent’s disease (MIM 300009) [2], confirming the suspected diagnosis of Dent’s disease 1. To date, our patient has a stable serum creatinine circa 3.0 mg/dL, maintaining normokalemia and absence of new episodes of symptomatic lithiasis with vigorous hydration and oral potassium citrate. 

During follow-up, it came to our attention that a 32-year-old male submitted to kidney transplantation and under regular follow-up at our center was a first-degree cousin of the aforementioned patient. The two patients’ mothers were sisters, both without any phenotypic changes. He presented with polyuria and polydipsia during childhood, without any described anomalies in routine bloodwork. At the age of 19, a serum creatinine of 1.46 mg/dL was detected, but no further evaluation was performed. He was hospitalized in April 2015 for acute-on-chronic kidney disease (serum creatinine 2.8 mg/dL) and hypokalemia. At the time, only mild proteinuria was detected (229 mg in 24-hour urine collection). Ultrasound showed only slightly reduced kidney size and cortico-medullary differentiation, without lithiasis. He was submitted to a kidney biopsy, which revealed glomerular hypertrophy, glomerulosclerosis, thickening of the tubular basement membranes, and chronic tubulointerstitial nephritis. Genetic testing for the most common mutations associated with nephronophthisis was requested, but no gene mutations were identified. After the hospital discharge, he maintained regular follow-up in nephrology consults. The kidney disease progressed, and an elevation of serum creatinine was noted over time, leading to the need for hemodialysis in September of 2019. He underwent deceased donor kidney transplant at our center in December of 2019. When his first-degree cousin was diagnosed with Dent’s disease, a genetic test was performed, which identified the same variant in the *CLCN5* gene (c.810C>G, p.(Ser270Arg)). 

## Discussion 

Historically, a group of X-linked recessive syndromes associated with hypercalciuric nephrolithiasis were described, all presenting with an underlying mutation in CLC-5 chloride transporter encoded by the *CLCN5* gene: X-linked recessive nephrolithiasis, X-linked recessive hypophosphatemic rickets, and LMW proteinuria with hypercalciuria and nephrocalcinosis [3]. Currently, this heterogenous group is referred to as Dent’s disease, an infrequent disorder documented in ~ 250 families worldwide [1], which is characterized by proximal tubule dysfunction and LMW proteinuria, associated with hypercalciuria, nephrolithiasis, nephrocalcinosis, and kidney failure [4, 5], all of which are present in our patient. LMW proteinuria is a cardinal manifestation of the disease, due to the excretion of α1-microglobulin and β2-microglobulin, retinol-binding protein and vitamin D-binding protein as well as being present in almost all affected males, but less frequently in females [6]. Approximately 30 – 80% of afflicted male individuals develop end-stage kidney failure, generally between the 3^rd^ and 5^th^ decades of life [4]. Mutations in *CLCN5* gene, located on the short arm of chromosome X, encoding for CLC-5, a Cl-/H+ antiporter, are present in 50 – 60% of the patients with Dent’s disease (Dent disease 1) [7]. 15% of the patients with Dent’s disease have a mutation in the *OCRL1* gene – located on the long arm of the chromosome X, which encodes for a phosphatase – and present with extra-renal manifestations. This entity is referred to as Dent’s disease 2 [8]. The remaining 25 – 35% patients possess neither of the aforementioned mutations but are thought to present defects in other genes not yet identified [6]. CLC5 is predominantly expressed on endosomes in the proximal tubule cells [9]. The mutation in *CLCN5* gene and consequent transporter anomaly lead to impaired acidification of the endosomes, resulting in defective endocytosis and dysfunctional proximal tubule cells. Thus, Dent’s disease may manifest as LMW proteinuria, hyperphosphaturia, aminoaciduria, glycosuria, hypercalciuria, and nephrocalcinosis [10, 11]. 

There is significant intra- and inter-familial variability of clinical manifestations, with no apparent correlation between genotype and phenotype [4]. The diagnosis of Dent’s disease should be suspected in individuals presenting with the following three characteristics: (a) LMW proteinuria, typically urinary protein/creatinine ratio > 0.6 g/g with albumin representing < 30% of the proteins lost in the urine, (b) hypercalciuria (> 4 mg/Kg in a 24-hour urine collection or > 0.25 mg of calcium/mg of creatinine in a spot urine sample, (c) at least one of the following: nephrocalcinosis, nephrolithiasis, hematuria, hypophosphatemia, or CKD [1]. The diagnosis may be confirmed by an inactivating mutation in *CLCN5* or *OCRL1*, but, as previously mentioned, there are a fraction of patients with Dent’s disease without mutations in these genes. In our first case, the clinical and analytical findings set us on a path of further etiological evaluation and attempts to reduce the risk of lithiasis, which led us to discovering medullary nephrocalcinosis and the patient’s enhanced sensibility to thiazide diuretics which, in itself, is not very usual. Our second patient was diagnosed with CKD at an earlier stage, describing only polydipsia and polyuria, without other features suggestive of Dent’s disease, such as non-albuminuric proteinuria or hypercalciuria. This heterogeneity in the presentation of this entity, as portrayed by our two patients, is a testament to the high degree of suspicion required to establish the diagnosis. The definitive diagnosis was achieved with genetic testing, which revealed a single nucleotide variant linked to Dent’s disease. As we mentioned before, there appears to be no correlation between genotype and phenotype, as shown by the stark difference of our patients’ natural history. 

Kidney biopsy is not helpful in the diagnosis of Dent’s disease because histological findings are nonspecific, ranging from normal to focal glomerulosclerosis [12]. As described, our second patient had been submitted to a kidney biopsy, which, unfortunately, did not aid in establishing a definitive diagnosis. 

The differential diagnosis should include other etiologies of proximal tubule dysfunction. Fanconi syndrome refers to the generalized dysfunction of the proximal tubule and can be secondary to genetic causes, such as Dent’s disease, Lowe syndrome, cystinosis, galactosemia, or Wilson’s disease, or acquired conditions such as drugs (mainly ifosphamide, cisplatin, anti-retrovirals, or aminoglycosides), heavy metals, or multiple myeloma [13]. The onset of symptoms at a young age was more suggestive of a genetic condition. Lowe syndrome and Dent’s disease 2 are caused by mutations on *OCRL1*, but the former is associated with short stature as well as ocular and cognitive abnormalities, features not present in Dent’s disease. One other aspect to consider in the differential diagnosis of the first patient was the presence of nephrocalcinosis. Nephrocalcinosis is defined by the deposition of calcium in the parenchyma and tubules and is caused by multiple entities. Moreover, medullary nephrocalcinosis is far more common than cortical nephrocalcinosis, increasing the complexity of determining the underlying cause [14]. 

To date, there are no specific therapies capable of changing the natural history of the disease. While many patients with nephrocalcinosis do not progress to end-stage kidney disease, patients with nephrocalcinosis in the context of Dent’s disease are more frequently associated with progression to end-stage kidney disease. Only supportive treatment can be offered, with an emphasis on prevention of nephrolithiasis [1]. Along with reduction of sodium intake, thiazides may be used to reduce calciuria, although patients with Dent’s disease are especially prone to adverse events, such as hypovolemia/hypotension and hypokalemia [15], as described in our patient. We found no data concerning therapies for correction of hypokalemia in these patients. In our first patient, given the hypotensive episodes after initiating a thiazide diuretic, we decided to initiate potassium supplementation, rather than a potassium-sparing diuretic such as spironolactone. Reduction in dietary calcium ingestion is not recommended, since it may aggravate bone disease, and low calcium intake is related to an increase in nephrolithiasis in the general population [16]. Given the tubular origin of the proteinuria, it is not clear if angiotensin-converting enzyme inhibitors are beneficial, but data is scarce [17]. Kidney transplantation seems effective, since it provides the recipient with proximal tubule cells containing CLC-5, and no recurrences of Dent’s disease have been documented [4]. 

In conclusion, although Dent’s disease is rare, it should be suspected in individuals with CKD of unknown etiology, LMW proteinuria, and hypercalciuria or nephrocalcinosis. While the progression to end-stage kidney disease in males is rather frequent, kidney transplant appears to be a good option in these patients. Regarding the genetic transmission of the variant present in chromosome X, it will be present in heterozygosity in female descendants, and, therefore, referral to a genetic consultation and counselling is recommended for the patient and family members at risk. 

## Funding 

None to declare. 

## Conflict of interest 

None to declare.
